# Mitogenome Phylogeny Including Data from Additional Subspecies Provides New Insights into the Historical Biogeography of the Eurasian lynx *Lynx lynx*

**DOI:** 10.3390/genes12081216

**Published:** 2021-08-06

**Authors:** Deniz Mengüllüoğlu, Hüseyin Ambarlı, Axel Barlow, Johanna L. A. Paijmans, Ali Onur Sayar, Hasan Emir, İrfan Kandemir, Heribert Hofer, Jörns Fickel, Daniel W. Förster

**Affiliations:** 1 Leibniz Institute for Zoo and Wildlife Research, Alfred-Kowalke-Str. 17, 10315 Berlin, Germany; hofer@izw-berlin.de (H.H.); Fickel@IZW-Berlin.de (J.F.); foerster@izw-berlin.de (D.W.F.); 2 Department of Wildlife Ecology and Management, Faculty of Forestry, Düzce University, Düzce 81620, Turkey; huseyinambarli@gmail.com; 3 School of Science and Technology, Nottingham Trent University, Clifton Lane, Nottingham NG11 8NS, UK; axel.barlow.ab@gmail.com; 4 Institute for Biochemistry and Biology, University of Potsdam, Karl-Liebknecht-Str. 24–25, 14476 Potsdam, Germany; paijmans.jla@gmail.com; 5 Department of Zoology, University of Cambridge, Downing Street, Cambridge CB2 3EJ, UK; 6 Department of Game and Wildlife, Cankiri Karatekin University, Cankiri 18100, Turkey; alionursayar@gmail.com; 7 Wildlife Department of General Directorate of Nature Conservation and National Parks, Turkish Ministry of Agriculture and Forestry, Ankara 06000, Turkey; hasanemir@gmail.com; 8 Department of Biology, Ankara University, Ankara 06000, Turkey; ikandemir@science.ankara.edu.tr; 9 Department of Biology, Chemistry and Pharmacy, Freie Universität Berlin, 10315 Berlin, Germany; 10 Department of Veterinary Medicine, Freie Universität Berlin, 10315 Berlin, Germany

**Keywords:** mitogenome, biogeography, intraspecific variation, Caucasian lynx, Balkan lynx, Himalayan lynx, Anatolian refugium

## Abstract

Previous molecular studies of the wide-ranging Eurasian lynx *Lynx lynx* focused mainly on its northern Palearctic populations, with the consequence that the reconstruction of this species’ evolutionary history did not include genetic variation present in its southern Palearctic distribution. We sampled a previously not considered Asian subspecies (*L. l. dinniki*), added published data from another Asian subspecies (*L. l. isabellinus*), and reassessed the Eurasian lynx mtDNA phylogeny along with previously published data from northern Palearctic populations. Our mitogenome-based analyses revealed the existence of three major clades (A: Central Asia, B: SE Europe/SW Asia, C: Europe and Northern Asia) and at least five lineages, with diversification in *Lynx lynx* commencing at least 28kyr earlier than hitherto estimated. The subspecies *L. l. isabellinus* harbors the most basal matriline, consistent with the origin of *Lynx lynx* in this subspecies’ current range. *L. l. dinniki* harbors the second most basal matriline, which is related to, and may be the source of, the mtDNA diversity of the critically endangered Balkan lynx *L. l. balcanicus*. Our results suggest that the Anatolian peninsula was a glacial refugium for Eurasian lynx, with previously unconsidered implications for the colonization of Europe by this species.

## 1. Introduction

The spatial distribution of terrestrial mammals is determined by the availability of suitable habitat, which has been shaped by historical climatic events and subsequently been modified by anthropogenic land use and land cover change [1]. A consequence of anthropogenic impacts on ecosystems is that a majority of extant terrestrial species that have wide geographic distributions today occur in fragmented and isolated populations (e.g., [2,3,4,5]). Such fragmentation and isolation enhances the spatial genetic structure generated by (historical) colonization and migration processes, with the consequence that populations harbor variation not present elsewhere [6,7]. In order to obtain a more complete understanding of the evolutionary history of such wide-ranging species, data from small, isolated populations, including those at range margins, must be considered in phylogeographic studies (southern Chinese tiger: [8]; Himalayan wolf: [9]; Himalayan brown bear: [10]). Such small or edge populations may harbor important components of a species’ total genetic diversity (clouded leopard: [11,12]; leopard cat [13]; Java sambar: [14]), particularly if refugial populations of former glacial periods have not substantially increased their range (brown bear in the Caucasus: [15]; brown bear in the Himalayas: [10]; Eurasian lynx in the Caucasus: [16]).

Among wild felids, the Eurasian lynx *Lynx lynx* has the widest distribution in the Palearctic. It inhabits a great variety of biomes with different climatic conditions and displays intraspecific variation in ecological requirements, morphology, and behavior [5], which is signified by six recognized subspecies [17]. Climatic shifts in Asia and climatic and anthropogenic factors in Europe have shaped the species’ genetic structure in its recent evolutionary history [18,19]. In Northern Asia, populations display clinal variation, while in Europe, recent population extirpation and isolation of small populations have created a heterogeneous, patchy genetic structure across the species’ distribution [19]. Historically, western Eurasian lynx populations were better connected and genetically more homogeneous, but in the past two centuries, anthropogenic factors disrupted the formerly existing connectivity among European populations [19,20]. As previous molecular studies on *L. lynx* focused mainly on European and Northern Asian populations [16,18,21,22,23,24,25,26,27,28,29], most information on the species’ phylogeography and evolutionary history derives from these northern Palearctic populations. However, during Pleistocene glaciations, species spreading towards Europe were stopped in their migration by extended ice sheets and remained in glacier-free southern refugia where they accumulated high genetic diversity [7,30,31]. These refugia were Iberia, Italy, and the Balkans [32], with the Balkans connecting eastward to Turkey and possibly to the Caucasus [7,15,33,34]. Such high genetic diversity was confirmed in *L. lynx* populations living in Anatolia [35,36,37] and in the greater Caucasus [16], which renders these populations very important for elucidating the diversity and evolutionary history of Eurasian lynx.

Furthermore, data from *L. lynx* populations from Central and Eastern Asia would also add essential information to the species’ evolutionary history. They live at the edges of the species’ range and were not as heavily impacted by climatic oscillations and anthropogenic factors as the northern Palaearctic populations, thus likely maintaining more of their ancestral genetic diversity [7,38]. While Eurasian lynx from Mongolia were already included in detailed phylogenetic analyses [18], specimens from other Central Asian (e.g., China and Turkestan) regions were not.

The Caucasian lynx *L. l. dinniki* populations live in a wide range of habitats and climatic zones, representing almost all European and Western Asian ecosystems ranging from warm Mediterranean to cold alpine regions. They live and reproduce in coniferous forests, and rocky mountains and steppes with no tree cover, and in contrast to their northern conspecifics that prey on ungulates (mostly roe deer), they prey almost exclusively on lagomorphs [39], which were confined to the same refugium during the Pleistocene glaciation [30]. Compared with other Eurasian lynx, individuals of *L. l. dinniki* are smaller, reflecting the plasticity of Eurasian lynx in ecological and behavioral traits [39,40,41].

The Himalayan lynx *L. l. isabellinus*, a subspecies that lives in extreme ecosystems such as high mountains and cold deserts, has already evolved adaptations to these harsh environments that distinguish it from other *L. lynx* populations in the north [42]. It lives in forested areas of the Pamir and Kunlun Mountains and in Central and Western China and preys on ungulates and lagomorphs, but it is also associated with alpine slopes above tree lines (up to 4200–4500 m a.s.l.) and high rocky areas of Central Asian deserts with scrub woodland and barrens, where it preys mostly on lagomorphs [42,43,44,45,46].

We therefore expect molecular data, in particular, from the Caucasian subspecies *L. l. dinniki* and the Himalayan subspecies *L. l. isabellinus* to add important information regarding the evolutionary history of Eurasian lynx. In contrast to the accumulated genetic knowledge about *L. l. dinniki* populations, similar knowledge is not available for *L.l. isabellinus.* The only molecular study available assessed neither its genetic diversity nor its position within the *L. lynx* phylogeny [38].

Regarding their mitochondrial diversity, Eurasian lynx matrilines have recently [18] been grouped into five haplogroups (HG), of which the most basal, HG1, was represented by *L. l. balcanicus*, which had split from the other European (*L. l. lynx, L. l. carpathicus*; HG2,3) and Northern Asian (*L. l. wrangeli*; HG3–5) subspecies ~96.5 thousand years ago (kya). In order to reassess the Eurasian lynx mtDNA phylogeny, we took this already existing dataset [18] and added six mitochondrial genomes (mitogenomes) of *L. l. dinniki* from three regions in Anatolia, Turkey, one published mitogenome from Northeast China (Khingan Mountains, [47]; *L. l. wrangeli* [17]), and one published mitogenome from Central China (Tianquan, [38]; *L. l. isabellinus* [17]). The inclusion of the two additional subspecies (*L. l. isabellinus*, *L. l. dinniki*) generated a new dataset that now includes mitogenomes of all six proposed *L. lynx* subspecies [17]. We discuss our results in the context of the historical distribution of European populations, including the critically endangered Balkan lynx (*L. l. balcanicus*), as well as in the context of inferring the evolutionary history of this wide-ranging felid species.

## 2. Materials and Methods

### 2.1. Sampling

**Figure 1:**
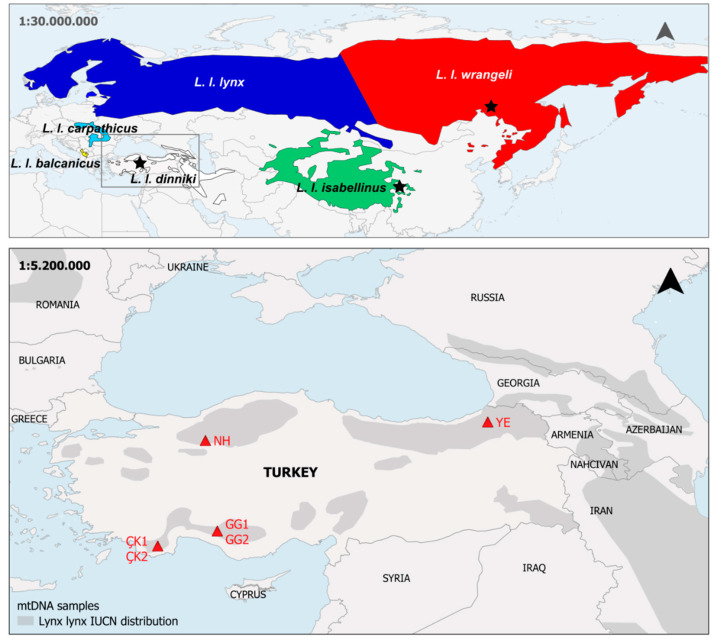
Top: Map of Eurasian lynx subspecies distribution. Stars indicate source area of mitogenomes added to the dataset in [18] (this study; [38,47]). Bottom: Map of Turkey depicting the locations of the four sampling sites for *L. l. dinniki* in Turkey. These sites are the Çığlıkara Forest Reserve (ÇK) and the Gidengelmez Mountains (GG) in Southwest Anatolia, the Nallıhan Mountains (NH) in Northwest Anatolia, and the Yusufeli-Kaçkar Mountains (YE) in the Lesser Caucasus. Shaded areas indicate the distribution of *Lynx lynx* [5].

Between 2012 and 2014, we collected Caucasian lynx (*L. l. dinniki*) fecal swabs from three regions of the species’ Anatolian distribution (Figure 1): the Çığlıkara Forest Reserve (ÇK) and the Gidengelmez Mountains (GG; both in Southwest Anatolia), the Nallıhan Mountains (NH; in Northwest Anatolia), and the Yusufeli-Kaçkar Mountains in the Lesser Caucasus (YE; in Northeast Anatolia). We also collected two *L. l. dinniki* hair samples, one from a zoo animal originating from Akseki, Antalya (near the Gidengelmez Mountains), and one from an individual from the Yusufeli-Kaçkar Mountains (Table 1).

### 2.2. DNA Extraction, Library Preparation, and Targeted Capture

We extracted DNA using a commercially available forensic DNA extraction kit (GEN-IAL GmbH, Troisdorf, Germany) following the manufacturer’s instructions. We then constructed Illumina sequencing libraries using 8 nt indices following a previously established protocol [48]. The libraries were enriched for mitochondrial sequences using hybridization capture. Generation of capture baits also followed an established protocol [49] by using three ~6 kb-long overlapping fragments of the Eurasian lynx mitogenome that were amplified using long-range PCR [50]. Due to the expected degraded nature of the samples’ DNA, we performed an in-solution targeted capture at 65 °C [50]. We then sequenced the mtDNA-enriched libraries using the 150-cycle Reagent Kit v3, generating 75 bp paired-end reads on an Illumina MiSeq (San Diego, CA, USA).

### 2.3. Bioinformatic Analysis

Paired-end reads were de-multiplexed using bcl2fastq v2.17.1.14 (Illumina, Inc. San Diego, CA, USA), followed by adapter sequences removal using cutadapt v1.3 [51]. The *minimum overlap length* parameter was set to 1, and *match-read-wildcards* was enabled. Quality trimming was carried out using the sliding window approach implemented in Trimmomatic [52], with the phred quality threshold set to Q = 20. Paired adapter-clipped and quality-trimmed reads were then merged using Flash v1.2.8 [53]. Sequences were then mapped to a Eurasian lynx reference mitogenome (GenBank acc. no. NC_027083.1) using BWA aln v0.7.10 [54] and samtools v1.19 [55] with default parameters (e.g., seed length of 32 bp, mismatch value of 0.04, reads removed with mapping quality < Q30). Duplicates were marked and removed with MarkDuplicatesByStartEnd.jar. Consensus sequences were generated and annotated in Geneious v8.1.7 [56], with ambiguous bases and bases with a read depth < 5 being masked with ‘Ns’. These were manually curated for start and stop codons in coding sequences (CDS) and inspected carefully for potential chimeras indicating Numts. Two repetitive regions of the control region were excluded from further analyses, as they could not be accurately resolved using short Illumina reads. 

### 2.4. Phylogenetic Analysis

We aligned the sequenced mitogenomes of the Anatolian *L. l*. *dinniki* samples (*n* = 6, this study) with 98 Eurasian lynx mitogenome sequences that had previously been published. Ninety-six were from a mitogenome population dataset, covering lynx populations across a wide range of the species’ distribution (NCBI PopSet ID: 1799628116; [18]), and two came from individuals sampled in China (one from Tianquan, Central China, acc. no. MH706704, [38]; one from the Khingan Mountains, Northeast China, acc. no. KR919624, [45]). The alignment consisted of 104 sequences and had a length of 16,449 nucleotides (without repetitive sequences in the control region; see above).

The mitochondrial phylogeny and coalescence times were estimated with BEAST v1.8.2 [57], using a coalescent tree prior and assuming a constant population size over the time span of the tree. A strict clock model was used with the per lineage substitution rate fixed to 1.54 × 10^−8^ substitutions/site/year, following Lucena-Perez et al. [18]. An HKY nucleotide substitution model with γ-distributed rate heterogeneity among sites was also specified. All other parameters and priors were left at their default value. The MCMC chain ran for sufficient time to achieve convergence and adequate sampling of all parameters (ESS > 200), verified using the program Tracer v1.6 [58]. TreeAnnotator [59] was used to remove an appropriate number of burn-in trees, select the maximum clade credibility tree from the posterior sample, and scale node heights to the median of the posterior. The resulting tree was visualized in FigTree v1.4.4 (http://tree.bio.ed.ac.uk/software/figtree/ accessed 25 November 2020). 

A median-joining network of the 104 mitogenome sequences was constructed using PopArt v1.7 [60]. 

## 3. Results

Out of the twelve Anatolian samples, we recovered complete or nearly complete mitochondrial genomes for six samples with a ≥5× sequencing depth at each base (Table 2). The number of missing positions per sequence ranged from 0 to 1760 (mean = 475), and each sample had a distinct haplotype. 

### 3.1. General mtDNA Phylogeny

**Figure 2:**
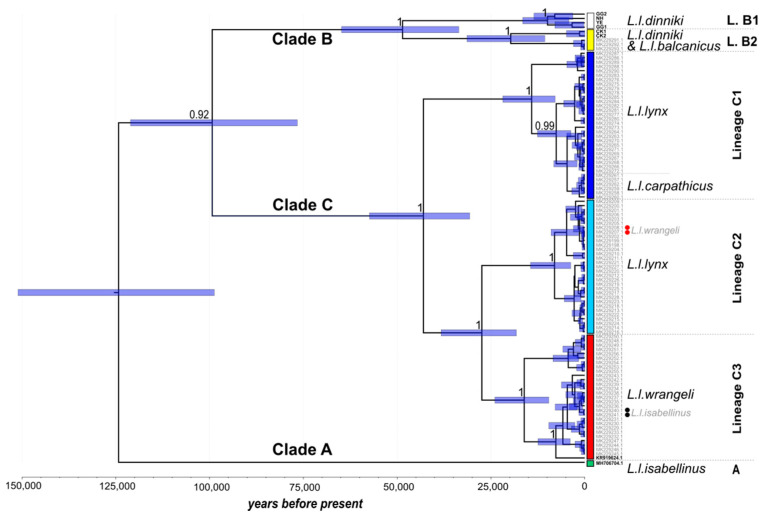
Bayesian phylogenetic tree of *L. lynx* mitogenome sequences. Posterior node support is given for all nodes with values >0.9. Blue bars at nodes indicate the 95% credibility intervals for coalescence times in years (*x*-axis). The three main branches are labeled according to clade assignment; lineage and subspecies assignment is indicated on the right. Vertical colored bars mark lineages, which are separated by dashed horizontal lines. Sequences are labeled by either GenBank accession number [18,38,47] or by location (*L. l. dinniki*). Red dots mark two *L. l. wrangeli* individuals within lineage C2 (see text for explanation); black dots indicate the two erroneously labeled *L. l. isabellinus* individuals in lineage C3.

Phylogenetic analysis of the alignment (104 sequences) revealed three well-supported clades (posterior support: 0.92–1), henceforth termed A, B, and C (Figure 2). Based on current subspecies delineation [17], Eurasian lynx clade A harbors one subspecies only, the Himalayan lynx *L. l. isabellinus* [38]. As this clade contains one sequence only, variability within this clade remains unknown. Clade B is formed by two subspecies: the Caucasian lynx *L. l. dinniki* and the Balkan lynx *L. l. balcanicus*. Clade C, by far the most variable of the three clades based on the sequences available, comprises three subspecies: the European lynx *L. l. lynx*, the Carpathian lynx *L. l. carpathicus*, and the Siberian lynx *L. l. wrangeli* (Figure 2). 

As we report new Eurasian lynx matrilines, including several basal ones, we adopt a new terminology based on a hierarchical structure of clades and lineages (Table 3) in order to avoid confusion with the previously published terminology [18]. 

### 3.2. History of mtDNA Clades and Lineages

*Clade A*: This basal clade (Figure 2, green) had diverged ~124 thousand years ago (kya; CI_95_: 151–98 kya) at the beginning of the Late Pleistocene from the most recent common ancestor (MRCA) of all three Eurasian lynx clades.

*Clades B and C*: About 25 ky later (~99 kya, CI_95_: 121–77 kya), clades B and C diverged from their MRCA. 

*Lineages B1 and B2*: About 49 kya (CI_95_: 74–34 kya), clade B split into two lineages: lineage B1 (Figure 2, white bar) and lineage B2 (Figure 2, yellow bar; corresponding to ‘haplogroup 1′ from [18], Table 3). The former contains sequences from *L. l. dinniki* only (*n* = 4), while the latter is formed by both *L. l. dinniki* (*n* = 2, bold font) and *L. l. balcanicus* (*n* = 3), rendering *L. l. dinniki* paraphyletic in this analysis. Within *L. l. dinniki*, lineage B1 is present in three sampling sites, while B2 is present only in one site (Çığlıkara Forest Reserve, in Southwest Anatolia). The divergence time between *L. l. dinniki* and *L.l. balcanicus* within lineage B2 is only ~20 ky (CI_95_: 31–11 ky).

*Lineages C1–C3*: About 43 kya (CI_95_: 57–31 kya), lineage C1 (Figure 2, dark blue bar) split from the MRCA of lineages C2 (Figure 2, bright blue bar) and C3 (red bar). Lineage C1 (corresponding to the former HG2 [18]) harbors both the European lynx (*L. l. lynx*) with individuals coming from Poland, Latvia, and the European part of Russia, and the Carpathian lynx (*L. l. carpathicus*). The latter individuals carry an *L. l. carpathicus*-specific haplotype that is not present in *L. l. lynx* individuals from lineage C1 (indicated in Figure 2). The youngest lineages to emerge were C2 and C3, which diverged about 27 kya (CI_95_: 38–18 kya) from their MRCA. Lineage C2 (former HG3 [18]) consists of two larger clusters, one exclusively formed by *L. l. lynx* mitogenomes from Scandinavia (Norway), and the second one predominantly formed by *L. l. lynx* mitogenomes from the European part of Russia (Kirov region, Ural). Thus, *L. l. lynx* appears in two lineages (C1 and C2), whereby not all descendants (*L. l. lynx* and *L. l. carpathicus*) share the same mitochondrial MRCA, rendering *L. l. lynx* mitochondrial lineages paraphyletic. The second cluster of lineage C2 contains two sequences (Figure 2, lineage C2, red dots) from individuals sampled in the Yakutsk region (Russian Far East; acc. nos.: MK229207, −208), which, based on their geographic origin and nDNA background, were assumed to be Siberian lynx *L. l. wrangeli* [16]. However, as these individuals carry *L. l. lynx* mtDNA but also *L. l. wrangeli* nuclear DNA, we consider them Siberian lynx introgressed with *L. l. lynx* mtDNA, which renders the mtDNA lineage C2 an *L. l. lynx*-specific lineage. Lineage C3 (former HGs 4 and 5 [18]) consists of lynx from only one subspecies as well: the Siberian lynx *L. l. wrangeli*. Within lineage C3, two individuals labeled as *L. l. isabellinus* (in [18]) were also included in our analysis (Figure 2, black dots). However, given their sampling locality (Ömnögovi, southern Mongolia), their assignment to the Himalayan lynx can be excluded [17]. We do not assume introgression of mtDNA into another subspecies here because the assignment to *L. l. wrangeli* was not solely based on mtDNA but was confirmed by nuclear DNA data that were also indicative for *L. l. wrangeli* [18]. Therefore, we consider lineage C3 to be an *L. l. wrangeli*-specific lineage. Another interesting observation regarding clade C is the onset of diversification in all three lineages ~15–10 kya (transition from Pleistocene to Holocene and end of glaciation), indicating rapid population expansion around that time. Finally, the presence of the two *L. l. wrangeli* individuals in lineage C2 (Figure 2, red dots), as mentioned above, would also render *L. l. wrangeli* (in addition to *L. l. lynx*) paraphyletic in this phylogeny.

**Figure 3:**
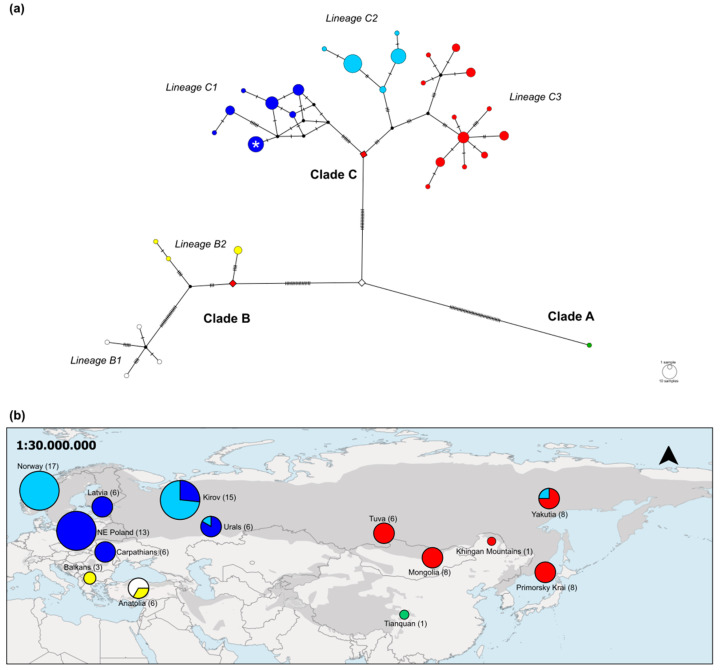
Relationships among *L. lynx* mitogenomes and their geographic distribution. (**a**) Median-joining network of *L. lynx* mitogenome haplotypes. Each circle represents a haplotype, with the circle size indicating the number of individuals sharing that haplotype. Black dots (vectors) were introduced by the network constructing algorithm and represent haplotypes not sampled. Dashes along lines that connect haplotypes indicate the number of nucleotide differences between haplotypes. Coloring of lineages follows Figure 2. Diamond-shaped symbols indicate the most recent common ancestors (MRCA), white: MRCA of all clades, red: MRCA for lineages. The white star in lineage C1 indicates the *L. l. carpathicus*-specific haplotype. (**b**) Geographic distribution of *L. lynx* mitogenomes across the Palearctic region. Coloring of circles follows Figure 2 and indicates clade/lineage assignment, circle size indicates number of individuals included. The figure was adapted from Lucena-Perez et al. [18] and extended to include *L. l. dinniki* (Anatolia, *n* = 6), *L. l. wrangeli* from China (Khingan Mountains, *n* = 1 [47]), and *L. l. isabellinus* (Tianquan, *n* = 1 [38]).

A large number of nucleotide differences corroborate the deep separation of the three Eurasian lynx clades, as visualized in the haplotype network (Figure 3a). The divergence appears most pronounced for clade A, with 35 mutations separating *L. l. isabellinus* from the putative most recent common ancestor of all three Eurasian lynx clades (Figure 3a, central white diamond). Yet, the branches leading to clades B and C also feature numerous mutations, exemplifying a fast accumulation of mutations within these lynx clades and lineages in the last 100 ky. Seventeen mutations separate the MRCA (Figure 3a, left red diamond) of lineages B1 (four haplotypes) and B2 (three haplotypes) from the putative Eurasian lynx MRCA haplotype, while 14 nucleotide changes separate the MRCA of all clade C lineages (24 haplotypes) from the putative Eurasian lynx MRCA haplotype (Figure 3a, right red diamond). The haplotype network shows a peculiar structure for lineage C1 (seven haplotypes; Figure 3a). This structure is not a matter of sample size because lineages C2 and C3 have similar sample sizes. However, while all other lineages (including B1 and B2) have either a tree-like or a star-like haplotype structure, lineage C1 (consisting of six *L. l. lynx* haplotypes and one *L. l. carpathicus* haplotype) deviates from this by having a cube-like structure.

## 4. Discussion

Our results indicate that southern Palearctic populations of Eurasian lynx harbor mitochondrial diversity not present in more northerly populations. Moreover, sampling of small populations at range margins in Anatolia (*L. l. dinniki*; this study) and China (*L. l. isabellinus*; [38]) revealed important relationships among matrilines in Eurasian lynx, contributing to our knowledge regarding more basal relationships within this species. 

These two subspecies (*L. l. dinniki* and *L. l. isabellinus*) not previously considered in phylogenetic analyses of complete mitogenomes are among the most basal in our phylogenetic reconstruction, together with *L. l. balcanicus* (Figure 2). Large areas of their distributions in the southern Palearctic are still unsampled, and their diversity is still largely uncharacterized (Figure 3b). As demonstrated here, targeted capture of mtDNA sequences from non-invasively collected sample material may be one approach to obtain such mtDNA data. 

### 4.1. Mitogenome Phylogeny

Clade A: The inclusion of the Himalayan lynx *L. l. isabellinus* into the matrilinear phylogeny of the Eurasian lynx revealed the presence of at least one additional (older) clade in the mtDNA variability of the species (Figure 2). It also shifted the previous age estimate for the first split within this species by approximately 28,000 years: from ~96.5 ky (CI_95_: 122–73 ky; [18]) to at least 124 ky (CI_95_: 151–98 ky). Unfortunately, clade A is represented by only a single individual in our analysis. Thus, we do not know the extent of variability within this clade. However, as clade A is basal in the Eurasian lynx phylogeny, we expect mtDNA variability to be large in this clade. In addition, its basal position in the phylogeny points to Central Asia as the likely origin for Eurasian lynx, supporting previous hypotheses [61]. We also included another Chinese *L. lynx* sample in our phylogeny, collected ~3200 km northeast of the sampling site of *L. l. isabellinus* [38]. The mitogenome of this sample (acc.no. KR919624; [45]) is placed in lineage C3 (Figure 2, acc.no. in bold; *L. l. wrangeli;* Figure 3b, Khingan Mountains), thereby indirectly confirming the Central Asian/Southern China distribution range of *L. l. isabellinus* [17].

Clade B: Inclusion of the Caucasian lynx *L. l. dinniki* did not change the inferred age of the divergence of clades B and C from their MRCA (Figure 2). Our estimate of 99 ky (CI_95_: 121–77 ky) is very similar to the previous estimate of 96.5 ky (CI_95_: 122–73 ky; [18]). The inclusion of *L. l. dinniki* had a striking impact on the relative position of *L. l. balcanicus* (basal ‘HG1’ in [18]). While the inclusion of *L. l. isabellinus* (clade A) caused the Balkan lynx to lose its basal position in the phylogeny, the inclusion of *L. l. dinniki* shifted the position of *L. l. balcanicus* further. The new phylogeny shows a deep ~49-ky-old split in clade B (the deepest within any clade) leading to the emergence of clade B lineages B1 and B2 (Figure 2). The striking change in the placement of *L. l. balcanicus* is that instead of comprising its own separate lineage, it diverged *within* lineage B2 from *L. l. dinniki* approximately 20 kya, shortly after the last glacial maximum (LGM: ~22 kya; [7]). There are three possible scenarios to explain these new results.

- *Scenario 1*: The Balkan lynx is a true subspecies, but the three Balkan lynx included in our analysis (from [18]) were descendants of a former hybridization event between *L. l. balcanicus* (male) and *L. l. dinniki* (female) and thus introgressed with *L. l. dinniki* lineage B2 mtDNA. Such introgressions occurred in other species, particularly if conspecific partners were scarce [62,63,64].
- *Scenario 2*: The entire lineage B2 is actually an *L. l. balcanicus* lineage that emerged ~48 kya from an MRCA with *L. l. dinniki* and had split into two sublineages ~20 kya, whereby ancestors of the two *L. l. dinniki* individuals from lineage B2 (ÇK1 and ÇK2; Figure 2) became introgressed with mtDNA from one of the two *L. l. balcanicus* sublineages. It needs to be noted that these two subspecies live on either side of the Bosporus junction between Asia (i.e., *L. l. dinniki*) and Europe (i.e., *L. l. balcanicus*), a junction that, during the evolution of these lineages, could have been traversed by either subspecies across the land masses connecting Asia and Europe via the Dardanelles and the Bosporus [65,66].
- *Scenario 3*: The Balkan lynx is not a true subspecies but belongs to *L. l. dinniki* [18,38].
- Even though the three scenarios differ in their parsimony, with scenario 3 being the most parsimonious, all three are feasible in principle and consistent with the data.

Clade C: Shortly after clade B had diverged into two lineages (~48 kya), clade C also diverged (~43 kya). There are two peculiarities to this clade. The first is that lineage C1 harbors two subspecies, namely, *L. l. lynx* and *L. l. carpathicus*, while the other two lineages of this clade, lineages C2 (*L. l. lynx*) and C3 (*L. l. wrangeli*), consist of single subspecies only (neither considering the introgressed *L. l. wrangeli* in lineage C2, nor the wrongly ‘*L. l. isabellinus*’-labeled lynx in lineage C3). All six Carpathian lynx included in lineage C1 shared the same subspecies-specific haplotype (Figure 3a). This Carpathian lynx haplotype has an estimated age of 5 ky only (CI_95_: 8–2 ky; Figure 2), a period too short for subspeciation even when considering that this estimate is based on just a single haplotype. This age is also much younger than the onset of diversification in the three clade C lineages (15–10 kya). A plausible explanation for the young age of this haplotype is an *L. l. lynx* origin. As the analysis of ancestral nuclear genotypes demonstrated Carpathian lynx-specific nDNA genotypes without signs of admixture [18], such an introgression scenario seems very likely. As Carpathian lynx form a small and isolated population [67] that is of high conservation value, it needs to be determined if introgression is a problem for this lynx subspecies as it is for the Scottish wildcat *Felis silvestris* [68].

The second peculiarity of clade C is the mitogenome distribution of *L. l. lynx*. The European lynx is the only *L. lynx* subspecies with mitogenomes occurring in two lineages (C1 and C2) with considerable divergence (~43 ky), the unresolved subspecies assignment in lineage B2 notwithstanding (see above). For *L. l. lynx,* three ancestral nDNA clusters have been reported [18], one of which occurs only in NE Poland (harboring mtDNA lineage C1), and another of which occurs only in Norway (harboring mtDNA lineage C2). The third nDNA cluster is present in Kirov and the Ural Mountains, where both mtDNA lineages (C1 and C2) are found. European lynx from Latvia included in the analysis (all belonging to mtDNA lineage C1) show an admixed nuclear genome between European lynx from Western Russia (Kirov, Ural) and NE Poland [18]. Thus, it is possible that mitogenomes of *L. l. lynx* from the European part of Russia have introgressed into the Central European *L. l. lynx* population. This scenario would result in a match between genotypes and haplotypes, emphasizing the distinctiveness of *L. l. lynx* in lineages C1 and C2, both in terms of their nDNA and their mtDNA.

### 4.2. Implications for European Populations of Lynx lynx

In the context of the published nuclear genomic data [18], our results indicate at least four colonization waves of Europe by Eurasian lynx. There was one southern wave by individuals of clade B and three northern ones by individuals of lineages C1 and C2 (we are not considering *L. l. carpathicus* here as we believe them to be introgressed with *L. l. lynx* mtDNA; see above). One of these northern waves led to the establishment of the Scandinavian population (i.e., Norway, lineage C2 only, HG3 in [18]), another one led to the establishment of *L. l. lynx* populations in Central Europe (i.e., NE Poland, lineage C1, HG2 in [18]), and the third one led to the establishment of populations in the European part of Russia and the Baltic states (i.e., Kirov, Ural, Latvia, lineage C1, HG2 in [18]). Lynx from the latter two waves formed a genotypic suture zone likely running through the Baltic region. 

Besides the detection of a new clade in the mtDNA phylogeny (clade A: *L. l. isabellinus*), the most striking result of the new analysis was the phylogenetic repositioning of *L. l. balcanicus*. Independent of the three scenarios outlined above, our study reveals a very close association of Balkan lynx with Caucasian lynx *L. l. dinniki* from Southwest Anatolia, thereby confirming analyses of mitochondrial cyt*b* and d-loop fragments that had previously suggested such association [35]. Clade B likely colonized Europe along a southern route. This route was probably via the Bosphorus junction, as it has been inferred for other species [15,33,69,70]. This could explain why the diversification in clade B preceded the one in clade C by ~5 ky (Figure 2), as post-glacial expansions in southern regions were possible earlier than in the northern regions.

The southern Palearctic contribution to European lynx populations is also highly relevant regarding the current and future status of the critically endangered Balkan lynx *L. l. balcanicus*. Thus far, the nuclear genomes of *L. l. dinniki* and *L. l. balcanicus* have not yet been compared, and thus the true status of the latter remains unresolved. In this regard, the Southwest Anatolian population of the Caucasian lynx is of particular interest, as this harbors the matriline (B2) present in the Balkan lynx sampled thus far. Future efforts to supplement *L. l. balcanicus* populations with lynx from elsewhere need to take the possibility of a partial southern Palearctic ancestry (i.e., *L. l. dinniki*) into account. 

### 4.3. Anatolia as a Hotspot of Diversity

Being positioned at the junction of Europe, Asia, and Africa, and having been influenced by glacial maxima only peripherally, Anatolia is a diversity hotspot for both endemic and widely distributed terrestrial organisms [71]. This diversity also extends to the genetic diversity within these species. In addition to the Eurasian lynx, for which multiple lineages and haplotypes are present in Anatolia, previous studies have demonstrated that Anatolia harbors extensive intraspecific diversity in many other terrestrial mammals, among them the brown bear [15], the gray wolf [72], the red fox [73], the marbled polecat [74], the stone marten [34], the ground squirrel [75], the brown hare [69,70], and the house mouse [76]. Molecular genetic studies of taxa in Anatolia thus provide valuable information about how biodiversity is generated and maintained in many species, as well as providing a framework to address current conservation needs for a host of threatened and endangered species (e.g., mountain and sand gazelles [77], leopard [78]).

## 5. Conclusions

Unfortunately, most studies on the ecology or the genetics of the Eurasian lynx have been conducted on populations in the northwestern (i.e., European) portion of its distribution, with a few exceptions [17,18,37,38,39,41,42,43,44,79]. Our mitogenome-based phylogeny reveals that two Asian subspecies that have gone relatively unstudied, *L. l. dinniki* and *L. l. isabellinus*, harbor two basal matrilines. This result is perhaps not unexpected considering an Asian origin of this species [61] and emphasizes the need to address the paucity of data for these two subspecies in order to improve our understanding of the evolutionary history and ecology of this species.

Of course, mitochondrial sequences are only a single genetic marker which does not capture the complexity of the evolution of the nuclear genome. This can lead to erroneous conclusions due to, for example, incomplete lineage sorting or introgression (e.g., [14,80]). We tried to avoid these pitfalls by considering introgression events in the interpretation of our results. We strongly encourage follow-up studies on *L. l. dinniki* and *L. l. isabellinus* employing nuclear genomic data, to be analyzed together with equivalent data available for the other subspecies.

## Figures and Tables

**Table 1:** Numbers and types of samples included in this study per study site.

Sampling Location	Area Protection Status	Fecal Swabs	Hair
Çığlıkara Forest Reserve (ÇK)	Research Forest	2	-
Gidengelmez Mountains (GG)	Wildlife Development Reserve	3	1 (zoo)
Nallıhan Mountains (NH)	Unprotected	3	-
Yusufeli-Kaçkar Mountains (YE)	Wildlife Development Reserve	2	1

**Table 2:** Summary of sequencing results following targeted capture for the six Anatolian samples with complete or near-complete mitogenomes.

Sample ID	Sample Type	Raw Reads	Joined Read Pairs after Quality Trimming	Duplication Percentage	Deduplicated Sequences	Percent Mitogenome Coverage at ≥5× Excl. Repetitive Region
NH	Fecal swab	4,212,534	1,613,945	94.02%	28,279	100
ÇK1	Fecal swab	1,682,350	557,712	84.29%	15,366	98.78
ÇK2	Fecal swab	1,797,014	569,045	78.95%	13,174	97.62
GG1	Fecal swab	11,647,032	4,271,917	98.20%	31,521	100
GG2	Hair (zoo)	1,215,728	349,044	86.96%	6727	91.53
YE	Fecal swab	3,518,972	1,101,722	96.16%	10,080	98.81

**Table 3:** MtDNA clades, lineages, and haplogroups of six Eurasian lynx subspecies ^a^ revealed in our study and what they correspond to in Lucena-Perez et al. [18].

Therminology Used in This Study	Subspecies Included: *Lynx lynx*	Origin	Corresponds to Haplogroup (HG) ^b^	Subspecies Included: *Lynx lynx*
Clade A	*isabellinus*	Southern China	-	*-*
Clade B	*dinniki, balcanicus*		HG 1	*balcanicus*
lineage B1	*dinniki*	Turkey (SW, NW, and NE Anatolia)	-	*-*
lineage B2	*dinniki, balcanicus*	Turkey (SW Anatolia), Montenegro, Serbia	HG 1	*balcanicus*
Clade C	*lynx, carpathicus, wrangeli*		HG 2–5	*lynx, carpathicus, wrangeli, isabellinus* ^c^
lineage C1	*lynx,*	NE Poland, Latvia, Russia (Kirov, Ural)	HG 2	*lynx, carpathicus*
	*carpathicus*	S Poland, Slovakia, Romania		
lineage C2	*lynx, wrangeli* ^c^	Norway, Russia (Kirov, Ural, Yakutia)	HG 3	*lynx, wrangeli* ^c^
lineage C3	*wrangeli*	Russia (Primorsky Krai, Yakutia, Tuva)	HG 4, 5	*wrangeli, isabellinus* ^d^
	*Isabellinus* ^d^	Mongolia (Ömgönovi)		

^a^: Subspecies assignment follows Kitchener et al. [17]. ^b^ [18]. ^c^ These two *L. l. wrangeli* individuals are likely introgressed with *L. l. lynx* mtDNA as their nuclear genomes are *L. l. wrangeli*. ^d^ Two lynx samples from Mongolia have been listed as *L. l. isabellinus* ([18]; Acc.no. MK229240, −41), but both mitogenome and WGS analysis assigned these samples to *L. l. wrangeli* [18]. Thus, the assignment of *L. l. isabellinus* to HG5 is erroneous. We included these two sequences in our analysis, in which they grouped with *L. l. wrangeli* (black dots in lineage C3, Figure 2).

## Data Availability

Sequences are deposited in GenBank under the following accession nos.: MZ672021-MZ672026.
